# A Dictionary of Terms Used in Medicine and the Collateral Sciences

**Published:** 1845-01

**Authors:** 


### A Dictionary of Terms used in Medicine and the Collateral Sciences. By *R. D. Hoblyn,* A.M. Sherwood, 1844. Second edition.

We hardly remember to have seen so much valuable matter condensed into such a small compass as this little volume presents. The first edition was published in 1835, and the present may be said to be almost re-written, introducing the most recent terms on each subject. The Etymology, Greek, Latin, &c., is carefully attended to, and the explanations are clear and precise.

In a large Appendix, Dr. H. has introduced several subjects of great utility to the student and practitioner. The first is headed "Affixes," in which are exhibited the principal *affixes or terminations* of words, in connexion with their compounds, which will be found to be a great aid to the memory. The next article is on Botany, and comprises a sketch of the artificial or Sexual System of Linnæus—also a sketch of the Natural System, and a Glossary of the adjective terms employed in Botany. The third article, on Climate, is abridged from the work of Sir James Clark. A Posological Table, carefully compiled, succeeds ; and then Materia Medica—Patent Medicines—Physiology—Toxicology—Vascular System—Weights and Measures—and, lastly, Zoology.

We cannot too strongly recommend this small and cheap volume to the library of every student and every practitioner.

